# Supplementary material for: Influence of Triazole Pesticides on Wine Flavor and Quality Based on Multidimensional Analysis Technology
Source: Molecules. 2020 Nov 28;25(23):5596. doi: 10.3390/molecules25235596 (PMC7730357; doi:10.3390/molecules25235596)
Supplement: Supplementary file 1 [file molecules-25-05596-s001.pdf]

**[Supplementary material]** <u>including 4 figures and 2 tables</u>

# Influence of triazole pesticides on wine flavor and quality based on multidimensional analysis technology

**Ouli Xiao [1,2,†], Minmin Li [3,†], Jieyin Chen [2], Ruixing Li [3], Rui Quan [3], Zezhou Zhang [1,2], Zhiqiang Kong [2,3,*] and Xiaofeng Dai [1,2*]**

1 Feed Research Institute, Chinese Academy of Agricultural Sciences, Beijing 100081, China; xiaoouli123@163.com (O.X.); zhangzezhou7689@163.com (Z.Z.)

2 State Key Laboratory for Biology of Plant Diseases and Insect Pests, Institute of Plant Protection, Chinese Academy of Agricultural Sciences, Beijing 100193, China; chenjieyin@caas.cn (J.C.)

3 Key Laboratory of Agro-Products Quality and Safety Control in Storage and Transport Process, Ministry of Agriculture and Rural Affairs/Institute of Food Science and Technology, Chinese Academy of Agricultural Sciences, Beijing 100193, China; liminmin@caas.cn (M.L.); liruixing06@163.com (R.L.); qr802319@163.com (R.Q.)

* Correspondence: kongzhiqiang@caas.cn (Z.K.); daixiaofeng_caas@126.com (X.D.);
Tel.: +86-10-62813566 (Z.K.); +86-10-62813566 (X.D.)

† These authors contributed equally to this work.

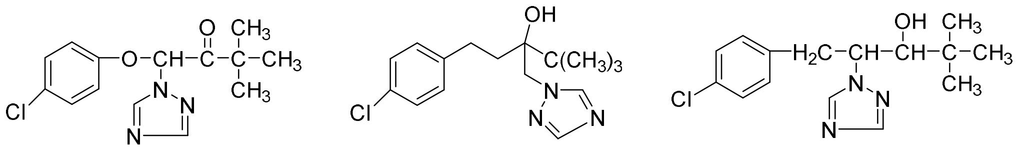

(a) (b) (c)

**Fig. 1.** Structure of triadimefon (a), tebuconazole (b), paclobutrazol (c).

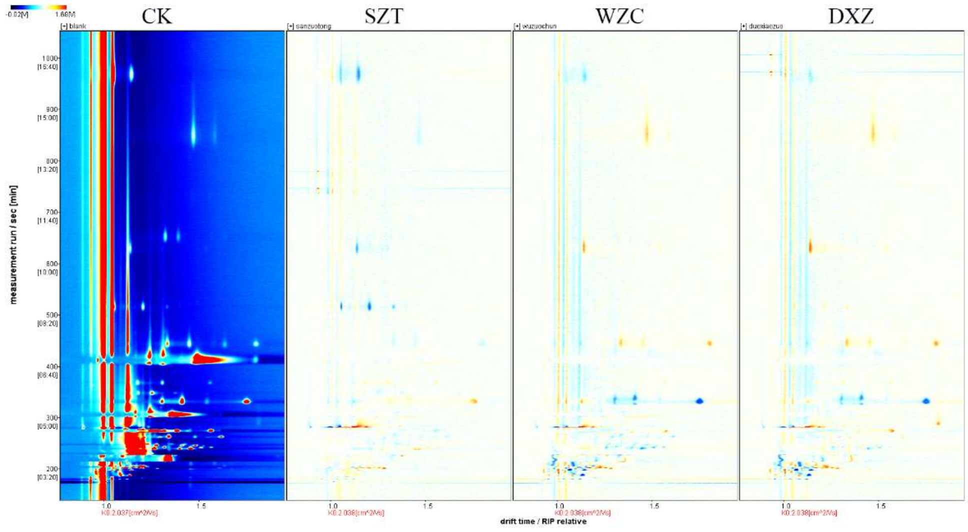


**Fig. 2.** GC-IMS spectra of volatile organic compounds in four groups of wine

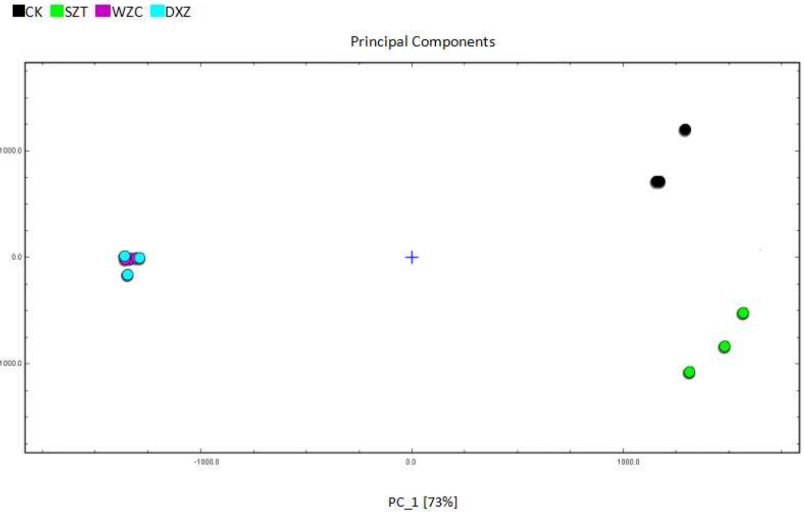


**Fig.3.** PCA (Principal Component Analysis) diagram of all samples.

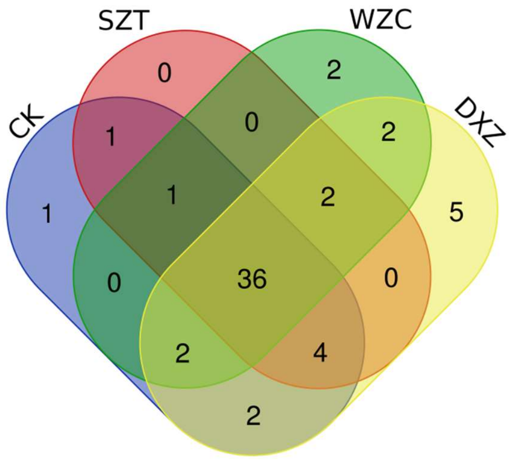


**Fig.4.** Veen diagram of the common volatile components in the four wine samples.

**Table 1.** Electronic nose principal component analysis table.

| | CK | SZT | WZC | DXZ |
|---|---|---|---|---|
| CK | - | 0.563 | 0.885 | 0.637 |
| SZT | 0.563 | - | 0.530 | 0.613 |
| WZC | 0.885 | 0.530 | - | 0.592 |
| DXZ | 0.637 | 0.613 | 0.592 | - |

The data in the table is the discrimination power value between the two samples, which higher than 0.5 indicating that there was a significant difference in flavor between the samples. “-” it is the same group of samples, not comparative

**Table 2.** Taste differences of wines

| Product names | Reference | Samples Distances | P Value |
|---|---|---|---|
| CK | DXZ | 194.18 | 0.00 |
| CK | SZT | 53.30 | 0.00 |
| CK | WZC | 54.92 | 0.00 |
| DXZ | SZT | 230.05 | 0.00 |
| DXZ | WZC | 166.65 | 0.00 |
| SZT | WZC | 72.74 | 0.00 |

P value of 0 means significant difference between the two groups
